# The effects of the current and past meteorological elements influencing the current pollen concentrations for different taxa

**DOI:** 10.1186/s40529-014-0043-9

**Published:** 2014-05-10

**Authors:** László Makra, Zoltán Csépe, István Matyasovszky, Áron József Deák, Zoltán Sümeghy, Gábor Tusnády

**Affiliations:** 1 Department of Climatology and Landscape Ecology, University of Szeged, P.O.B. 653, Szeged, HU-6701 Hungary; 2 Department of Meteorology, Eötvös Loránd University, Pázmány Péter st. 1/A, Budapest, HU-1117 Hungary; 3 Department of Physical Geography and Geoinformatics, University of Szeged, P.O.B. 653, Szeged, HU-6701 Hungary; 4 Mathematical Institute of the Hungarian Academy of Sciences, P.O.B. 127, Budapest, HU-1364 Hungary

**Keywords:** Climate change, Allergenic taxa, Pollen, Pollen season, Respiratory allergy, Factor analysis with special transformation

## Abstract

**Background:**

It is an important issue to separate the current and past components of the meteorological parameters influencing the current pollen concentration for different taxa. For this purpose a new statistical procedure, factor analysis with special transformation is introduced. The data set used covers an 11-year period (1997–2007) including daily pollen counts of 19 taxa and 4 climate variables (mean temperature, precipitation amount, global solar flux and relative humidity).

**Result:**

The taxa examined can be classified into three groups, namely arboreal deciduous (AD), arboreal evergreen (AE) and herbaceous (H) taxa. It was found that a better comparison can be established if the taxa are separated within each group according to the starting month of their pollen season. Within the group of AD taxa, *Alnus, Populus* and *Ulmus* are marked by a late summer – early autumn peak of the role of past meteorological elements exceeding the role of the current ones almost all over the pollen-free period. For *Juglans, Morus, Platanus* and *Quercus,* the major weights of the current meteorological elements in the spring and early summer show the most characteristic contribution to the pollen production. For AE taxa, the picture is no clear. For H taxa, the curves of *Cannabis, Plantago, Rumex* and *Urtica* indicate the most equalized course of weights. *Ambrosia, Artemisia* and Chenopodiaceae comprise the highest weights of the past weather conditions of all taxa until at least three months before the start of the pollination. Interactions between the phyto-physiological processes and the meteorological elements are evaluated.

**Conclusion:**

Separation of the weight of the current and past weather conditions for different taxa involves practical importance both for health care and agricultural production.

## Background

Recently, the earth’s ecosystem has been experiencing a global warming. Projections of future climate change suggest further global warming, sea level rise and an increase in the frequency of some extreme weather events (Parry et al. 2007). By the late 21st century, distributions of European plant species are projected to have shifted several hundred kilometres to the north (Parry et al. 2007; Lindner et al. 2010). The rate of change will exceed the ability of many species to adapt. As for plant phenology, the timing of seasonal events in plants is changing across Europe due to changes in the climate conditions. Between 1971 and 2000, the average advance of spring and summer was 2.5 days per decade. The pollen season starts on average 10 days earlier and is longer than it was 50 years ago (Feehan et al. 2009).

Climate change in association with an extended urbanization, with high levels of vehicle emissions in urban areas, living in artificial environment with little movement exert negative impact on individuals in most industrialized countries. Hence, these factors may contribute to explain the increasing frequency of respiratory allergy and asthma (D’Amato 2011). Pollen with other factors and microorganisms, like fungal spores is an important trigger of respiratory diseases. Both quantity related (total annual pollen counts, annual peak pollen counts) and phenological (start, end and duration of the pollen season) characteristics of different taxa are functions of the meteorological variables. Obviously there are other factors as well greatly influencing pollen attributes, like soil biology, topography, nutrient availability, etc. Greater concentrations of carbon dioxide and, consequently, higher temperatures may increase pollen quantity and induce longer pollen seasons (Ziska et al. 2003; Clot 2008). Pollen allergenicity can also increase as a result of these changes in climate. Furthermore, there is evidence that high levels of traffic-derived air pollutants may interact with pollen and bring about more intense respiratory allergy symptoms (Hjelmroos et al. 1999; Motta et al. 2006). Accordingly, global warming may induce a wide pollen-related public health problem; for which the societies should be prepared in time.

Predicting the pollen season characteristics (e.g. the start of the pollen season) as early as possible is of basic importance in order to prepare sensitive people in time for the days of pollen dispersion. Our analysis helps to identify those key periods, meteorological variables of which affect mostly the daily pollen concentrations of the given taxon. Changing climate involves different changes in the weather elements of the individual seasons. A potential importance of the procedure is that a period with a changing climate that may have a major impact on the current pollen counts can be detected by the past of meteorological elements.

The main aim of this paper is to study an extended spectrum of airborne pollen characteristics (19 plant taxa) for the Szeged region in Southern Hungary. A novel procedure is introduced in order to separate the current and past effects of the climate variables influencing the current pollen concentration. Namely, arrays of daily values of the influencing climate variables in the pollen season of the taxa examined were labelled as current variables, while arrays of daily values of these variables in the period starting from the first pollen-free day following the previous pollen season to the last pollen-free day preceding the actual pollen season were labelled as past variables. This kind of separation has not been demonstrated in the literature. Results are evaluated with special attention to the interactions between the phyto-physiological processes and the meteorological elements; furthermore, they are compared for each taxon based on different aspects.

## Materials and methods

### Location and data

**Figure 1:**
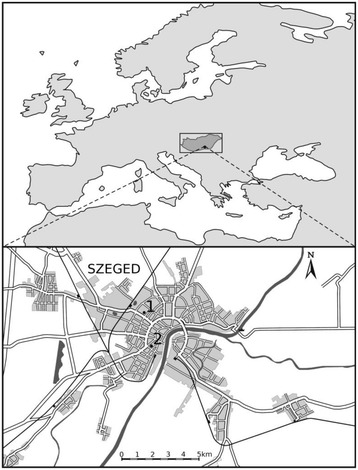
**Location of Europe including Hungary (upper panel) and the urban web of Szeged with the positions of the data sources (lower panel). 1**: meteorological station; **2**: aerobiological station. The distance between the aerobiological and the meteorological station is 2 km.

Szeged (46.25°N; 20.10°E), the largest settlement in South-eastern Hungary is located at the confluence of the Rivers Tisza and Maros (Figure 1). The area is characterised by an extensive flat landscape of the Great Hungarian Plain with an elevation of 79 m above sea level. The city is the centre of the Szeged region with 203,000 inhabitants. The climate of Szeged belongs to Köppen’s Ca type (warm temperate climate) with relatively mild and short winters and hot summers (Köppen 1931).

The data set consists of daily pollen counts (average daily pollen count per cubic meter of air) of 19 taxa taken over the 11-year period 1997–2007. With their Latin (English) names they are: *Alnus* (alder), *Ambrosia* (ragweed), *Artemisia* (mugwort), *Betula* (birch), *Cannabis* (hemp), Chenopodiaceae (goosefoots), *Juglans* (walnut), *Morus* (mulberry), *Pinus* (pine), *Plantago* (plantain), *Platanus* (plane), Poaceae (grasses), *Populus* (poplar), *Quercus* (oak), *Rumex* (dock), *Taxus* (yew), *Tilia* (linden), *Ulmus* (elm) and *Urtica* (nettle). These 19 taxa produce 93.2% of the total pollen amount for the given period. Taxa with the highest pollen levels include *Ambrosia* (32.3%), Poaceae (10.5%), *Populus* (9.6%) and *Urtica* (9.1%), which together account for 61.5% of the total pollen production (University of Szeged, Pollen Database, 1989–2007) (Table 1).

**Table 1:** **Plant habits and phenological pollen season characteristics**

**Taxa**	^**1**^ **Plant habit**	**Pollen season**		
		**Start**	**End**	**Duration, day**
*Alnus*	AD	Feb 3	Apr 8	65
*Betula*	AD	Mar 22	May 8	48
*Juglans*	AD	Apr 12	May 19	38
*Morus*	AD	Apr 18	May 22	35
*Platanus*	AD	Apr 8	May 20	43
^***2***^ ***Populus***	**AD**	Feb 27	Apr 20	53
*Quercus*	AD	Apr 2	May 11	40
*Tilia*	AD	May 16	Jul 1	47
*Ulmus*	AD	Feb 6	Apr 8	62
*Pinus*	AE	Apr 25	Jun 8	45
*Taxus*	AE	Feb 13	Apr 15	62
^***2***^ ***Ambrosia***	**H**	Jul 15	Oct 15	93
*Artemisia*	H	Jul 18	Oct 9	84
*Cannabis*	H	Jun 6	Sep 3	90
Chenopodiaceae	H	Jun 22	Oct 11	112
*Plantago*	H	May 12	Aug 29	110
^**2**^ **Poaceae**	**H**	Apr 16	Oct 11	180
*Rumex*	H	May 13	Aug 12	92
^***2***^ ***Urtica***	**H**	May 4	Sep 26	146

^1^AD: arboreal deciduous, AE: arboreal evergreen, H: herbaceous.

^2^Bold: taxa with the highest pollen levels in Szeged region.

As regards the taxa with the highest pollen concentrations, *Ambrosia* genus has only one species, namely *Ambrosia artemisiifolia* (Common Ragweed) in Szeged region that appears both in the urban environment and in the countryside. Ragweed occurs especially frequently west of the city. The ruling north-western winds can easily transport pollen into the city. Several species of Poaceae family, namely *Agropyron repens* (Common Couch), *Poa trivialis* (Rough Meadow-grass), as well as *Poa bulbosa* (Bulbous Meadow-grass) over untouched areas, furthermore *Poa angustifolia* (Narrow-leaved Meadow-grass) and *Alopecurus pratensis* (Meadow Foxtail) in floodplain and along the dyke surrounding Szeged region represent a substantial proportion in the city. Along the urban lakesides *Phragmites australis* (Common Reed) is the most frequent Poaceae. For *Populus* genus, natural species of *Populus alba* (White Poplar) and *Populus canescens* (Grey Poplar) are the most frequent in the city and are characteristic in floodplain forests along the Tisza and Maros Rivers. In addition, cultivated poplars such as I-273 Poplar and *Populus x euroamericana* (Canadian Poplar) and its variants are frequently planted in urban parklands, public places, as well as along roads in peripheries. *Urtica* genus with its only species of *Urtica dioica* (Common Nettle) in Szeged region is prevailing in the floodplain forest underwood of the Tisza and Maros Rivers, road- and channel-sides and in locust-tree plantations around the city. *Urtica* also occurs in neglected grassy lands of the city area (Deák 2010).

The remaining species seldom occur here. *Alnus* species are only found in the Botanical Garden of Szeged. Pollen of *Artemisia, Cannabis,* Chenopodiaceae and *Rumex* can come from neglected areas of both the city and its surroundings, as well as from stubble pastures. *Betula, Juglans, Pinus, Platanus, Taxus* and *Tilia* species have been planted exclusively in public places and gardens; they have no natural habitats in the Szeged region. However, since the 1960s *Pinus* (*Pinus sylvestris* and *Pinus nigra*) species have been extensively planted in the sandy regions north-west of Szeged within the framework of an afforestation programme. Their pollen can easily reach Szeged via north-western winds. *Morus* is planted along avenues and in public places. *Plantago* species occur in natural grassy areas of both the city and its surroundings. *Quercus* species are planted along the embankment surrounding the city, as well as north of the city. *Ulmus* is planted in the city too; however it is not very common there. Formerly planted *Ulmus pumila,* as an adventive species, also occurs, but its spread is not characteristic. Large natural stocks of *Ulmus minor* together with *Ulmus laevis* live in the oak-elm-ash alluvial forests alongside the River Maros where planted and spontaneous stocks also appear. They can be found spontaneously even in the willow-poplar alluvial forests thanks to the mature stocks of the Pécska forest on the Romanian side (Deák 2010).

The pollen content of the air was measured using a 7-day recording Hirst type volumetric spore trap (Hirst 1952) (Figure 1). Pollen sampling was performed as follows: A specific tape was made adhesive by washing it with silicone oil. The sampler absorbed air at a rate of 10 l/min (=14.4 m^3^/day, which is corresponding to the daily requirement of an adult person) and was supplied with a timer, to which a rotating drum was fitted. The drum moved the adhesive tape (2 mm/h) where pollen grains adhered. After a week of exposure, the tape was removed and cut to a length corresponding to 24 h pollen sampling, covered with a gel mounting agent containing fuxin as a stain and put on a microscope slide. Afterwards, the samples were examined under a light microscope at a magnification of 400× to determine pollen types and counts. Five horizontal sweeps were analysed on each slide. Horizontal sweeps were used because the variation in the concentration during the day can be observed along this axis (the direction of the tape shifts in the sampler). The accuracy of the measurement was proportional to the number of sweeps and the concentration of particles. Counting was performed using a standard sampling procedure. Pollen concentrations were expressed as number of pollen grains ⋅ m^−3^ of air (Käpylä and Penttinen 1981; Peternel et al. 2006). The air sampler is located on top of the building of the Faculty of Arts at the University of Szeged, approximately 20 m above the ground surface (Makra et al. 2010).

Time dependent daily pollen concentrations are influenced by numerous underlying processes. They include (1) genetic attributes, (2) soil type including location specific nutrient availability, (3) meteorological conditions in the root zone, (4) land use changes, (5) current and preceding weather variables, (6) the height of the planetary boundary layer (PBL) and the ventilation coefficient, (7) long-range pollen transport, (8) resuspenson of the pollen grains, (9) disruption of the pollen grains, and (10) pollen grains as condensation nuclei.

Though the above mentioned components can affect current pollen level, they are omitted and we analyse only (5), since our purpose is separating the effects of the current and past meteorological elements influencing the current pollen concentrations.

Nevertheless it should be mentioned that (2) indicates a definite weather dependence. Namely, plant growth and development largely depend on the combination and concentration of nutrients. Whilst, nutrient deficiency can have a significant impact on plant development, resulting in reduced plant quality or reduced pollen production. Nutrients are usually obtained from the soil through plant roots, but many factors like soil properties (chemistry, composition, compaction, humidity, temperature) and pH substantially influence the efficiency of nutrient acquisition (Forde and Lorenzo 2001; López-Bucio et al. 2003). The availability of certain nutrients for plants is largely weather dependent. From the above-mentioned soil parameters humidity and temperature are in direct, while the remaining components are in indirect association with climate variables.

Since some of the above parameters are either constant for a given taxon (1), or can be neglected (4), or not available (3, 6) or are hard to model (2, 8, 9) or difficult to consider their effect when assessing the target variable (7, 10), they are omitted from further consideration. At the same time, daily values of the current and past weather variables [mean temperature, (T,°C); relative humidity (RH,%), global solar flux (GSF, Wm^−2^) and precipitation amount (P, mm) influencing daily pollen concentrations were used in the study. They were collected in a meteorological station located in the inner city area of Szeged (Figure 1). These elements were used since they indicate the highest impact on pollen production (Galán et al. 2000; Bartková-Ščevková 2003; Štefanič et al. 2005; Kasprzyk 2008; Makra and Matyasovszky 2011).

The pollen season is defined by its start and end dates. For the start (end) of the season we used the first (last) date on which 1 pollen grain m^−3^ of air is recorded and at least 5 consecutive (preceding) days also show 1 or more pollen grains m^−3^ (Galán et al. 2001). For a given pollen type, the longest pollen season during the 11-year period was considered for each year.

Daily values of mean temperature, relative humidity, global solar flux and precipitation amount were labelled as current meteorological conditions for the pollen season of a given taxon, while they were considered as past meteorological conditions for the period starting from the first pollen-free day following the previous pollen season to the last pollen-free day preceding the actual pollen season.

### Factor analysis with special transformation

Factor analysis identifies any linear relationships among subsets of examined variables and this helps to reduce the dimensionality of the initial database without substantial loss of information. First, a factor analysis was applied to the initial datasets consisting of 9 variables (8 explanatory variables including 4 climatic variables in the past and the same 4 climatic variables on the actual day, and 1 resultant variable defined by the daily pollen concentration of the given taxa) transforming the original variables to fewer variables. These new variables (called factors) can be viewed as latent variables explaining the joint behaviour of past and current meteorological elements as well as current pollen concentration variables. The number of retained factors can be determined by different criteria. The most common and widely accepted one is to specify a least percentage (80%) of the total variance in the original variables that has to be achieved (Jolliffe 1993; Liu 2009). After performing a factor analysis, a special transformation of the retained factors was made to discover to what degree the above-mentioned explanatory variables affect the resultant variable and to give a rank of their influence (Jahn and Vahle 1968). When performing factor analysis on the standardized variables, factor loadings received are correlation coefficients between the original variables and, after rotation, the coordinate values belonging to the turned axes (namely, factor values). Consequently, if the resultant variable is strongly correlated with the factor (the factor has high factor loading at the place of the resultant variable) and - within the same factor - an explanatory variable is highly correlated with the factor, then the explanatory variable is also highly correlated with the resultant variable. Hence, it is advisable to combine all the weights of the factors together with the resultant variable into one factor. Namely, it is effective to rotate so that only one factor has big weight with the resultant variable. The remaining factors are uncorrelated (are of 0 weights) with the resultant variable (Jahn and Vahle 1968). This latter procedure is called special transformation.

**Figure 2:**
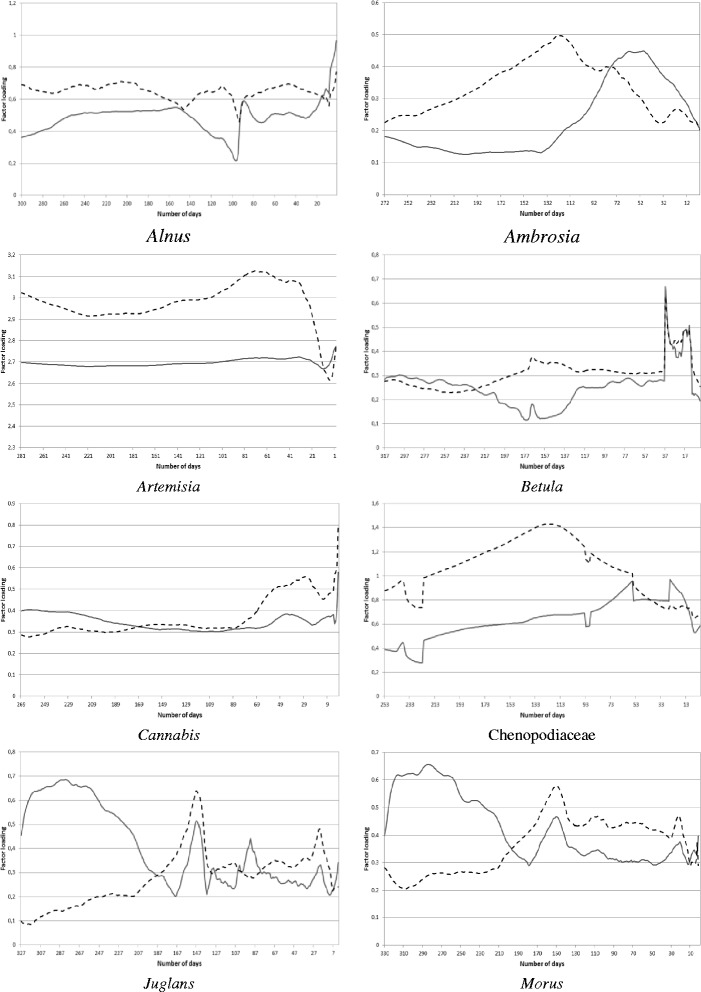
**Total weights of the factor loadings for the current (solid line) and past (dashed line) meteorological elements influencing the current pollen concentrations for** ***Pinus, Plantago, Platanus,*** **Poaceae,** ***Populus, Quercus, Rumex*** **and** ***Taxus.***

**Figure 3:**
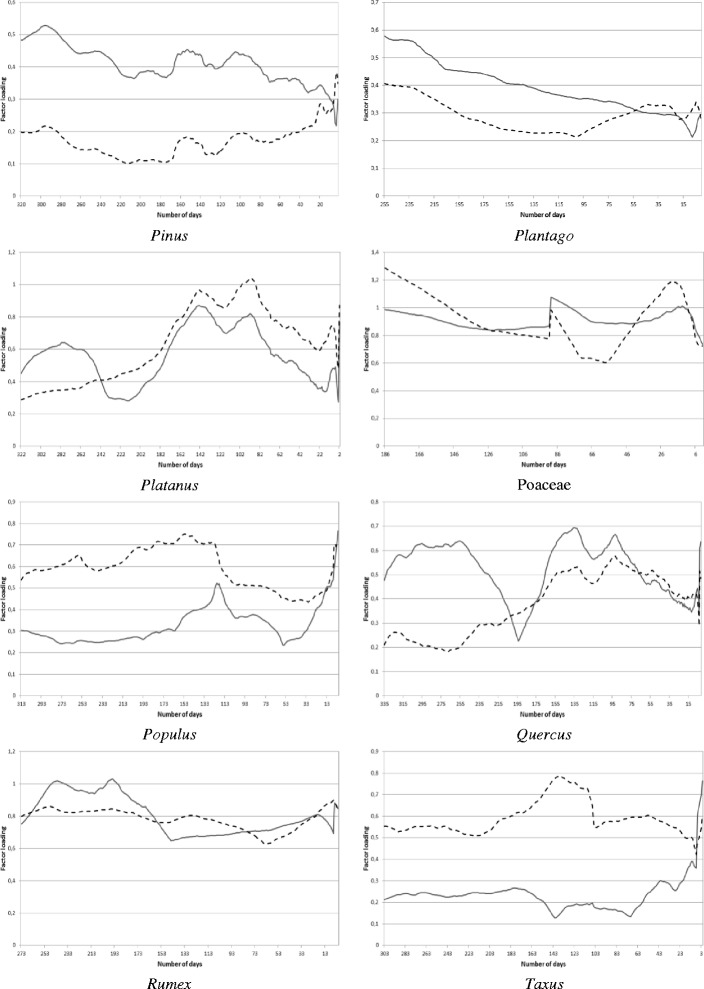
**Total weights of the factor loadings for the current (solid line) and past (dashed line) meteorological elements influencing the current pollen concentrations for Alnus, Ambrosia, Artemisia, Betula,** ***Cannabis,*** **Chenopodiaceae,** ***Juglans*** **and** ***Morus.***

**Figure 4:**
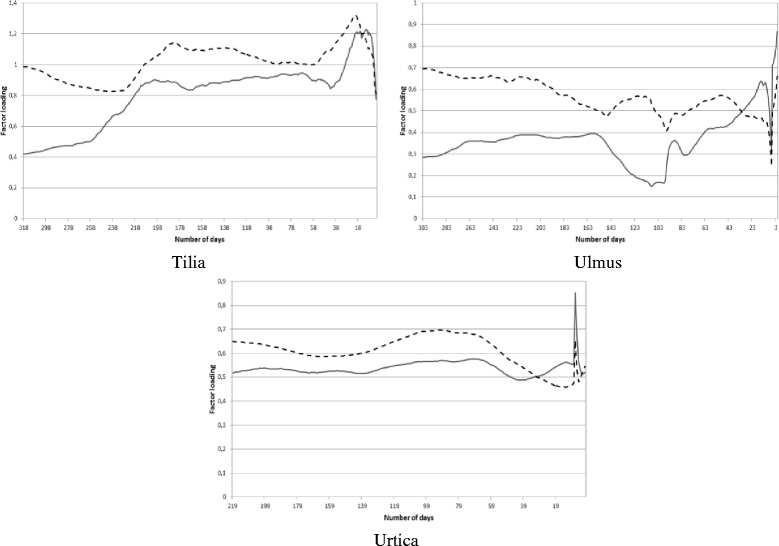
**Total weights of the factor loadings for the current (solid line) and past (dashed line) meteorological elements influencing the current pollen concentrations for** ***Tilia*** **,** ***Ulmus*** **and** ***Urtica.***

The calculation procedure and main results are presented in detail for Poaceae, because this species has the longest pollen season in Hungary and has big contribution to the total annual pollen production. In order to assess the effect of the antecedent and current meteorological conditions on the current Poaceae pollen concentration, the 1st-day, 2nd-day, …, 180th-day values of both the pollen concentration and the four meteorological elements of the current pollen season were taken. [The duration of the Poaceae pollen season in Szeged lasts from April 16 until October 12, namely 180 days. (Table 1)]. The values of these meteorological variables were then cumulated for 186-day, 185-day, …, 1-day periods starting 186 days, 185 days, …, 1 day before the actual day of the actual pollen season. This is because there are 186 days between the end of the previous-year pollen season and the beginning of the actual pollen season. Hence, 186 9-dimensional data sets were produced and a factor analysis with special transformation was performed for each of them (Figure 2). The altogether 5496 factor analyses carried out for all the 19 taxa resulted in 3 and 4 factors, except for *Artemisia* (2 factors) (Figures 2, 3, 4).

The reason of using factor analysis with special transformation is that this procedure makes possible to determine the weight and the rank of importance of the individual variables influencing the target variable, namely the current daily pollen counts. At the same time, when using autocorrelation, time dependent associations with the target variable can be determined; however, the weight of the individual influencing variables in the current pollen counts cannot be specified.

## Results

When analyzing the results, note that if the total weight of the factor loadings for the current climate variables is higher (lower), accordingly its effect is also higher (lower) than that of the past climate variables.

**Figure 5:**
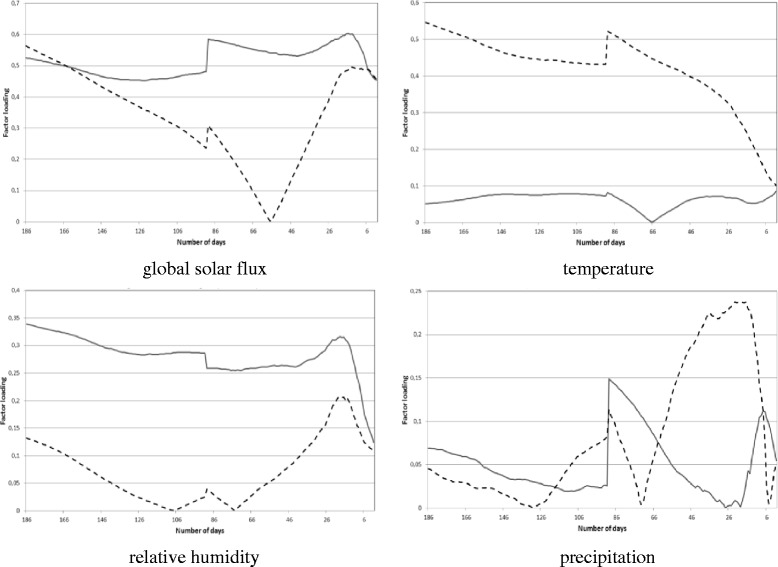
**Weights of the factor loadings for the current (solid line) and past (dashed line) meteorological elements influencing the current pollen concentrations for Poaceae.**

The curve of the total weights of the factor loadings for the current and past meteorological elements (Figures 2, 3, 4) were associated with the curve of the weights of the factor loadings for the current and past meteorological elements (Figure 5). The purpose of this relationship was to detect the weight of the current/past effect of the climate parameters back in time having the highest role in determining the current pollen counts.

For *Alnus,* the weights of the past meteorological parameters are somewhat higher with small variability compared to those of the current ones almost in the whole pollen-free season (Figure 3, 1st panel from above, left). For *Ambrosia*, the total weights of the factor loadings for the past meteorological variables are gradually increasing from the first pollen-free day following the last *Ambrosia* pollen season until the middle of March (Figure 3, 1st panel from above, right). For *Artemisia,* the past meteorological elements have a much more important effect on pollen production almost in all the pollen-free period, especially between mid-April and mid-June (Figure 3, 2nd panel from above, left). For *Betula,* the effect of the past and current meteorological elements is very similar in the preceding pollen-free season, except the period from August until the end of the autumn when the difference between the weights of the past and current meteorological parameters is the highest with the bigger weights of the past elements (Figure 3, 2nd panel from above, right). For *Cannabis,* the past and current weather conditions have a balanced role, i.e. almost equal weights in the pollen-free period (Figure 3, 3rd panel from above, left). For Chenopodiaceae family, after finishing the previous pollen season (October 11), the weights of the past meteorological elements are substantially higher than those of the current ones. Whilst, around two months before the current pollen season this difference vanishes (Figure 3, 3rd panel from above, right). For *Juglans* and *Morus,* with very similar curves, after finishing the previous pollen season, the weight of current meteorological elements is higher reaching the maximum weight in early July, while afterwards the difference practically disappears (Figure 3, 4th panel from above, left and right). *Pinus* and *Plantago* genera are sensible especially for the current weather conditions (Figure 2, 1st panel from above). The past meteorological elements indicate the smallest effect on these taxa of all analyzed in the study. For *Platanus,* the weights of the current meteorological elements exceed those of the past climate in the summer period (late May – early August), however their values are very similar to each other (Figure 2, 2nd panel from above, left). For For Poaceae the results are in agreement with our preliminary expectation. Namely, current climate has a higher importance close to the current pollen release; while back in time the role of both the past and current climate varies depending on periods (Figure 2, 2nd panel from above, right). *Populus,* the past meteorological elements have higher weights compared to the current climate parameters except 2–3 days before the start of the pollen season (Figure 2, 3rd panel from above, left). For *Quercus,* the weight of the current meteorological elements is much higher than that of the past climate parameters from mid-May until mid-August.. However, in the remaining period these values are very similar (Figure 2, 3rd panel from above, right). For *Rumex,* only very small differences can be observed in the weights of the current and past meteorological elements in the pollen-free period preceding the current pollen season (Figure 2, 4th panel from above, left). For *Taxus,* the past meteorological elements have a prominently remarkable role between in the pollen-free period except for a few days before the start of the current pollen season in early February when the current meteorological elements become dominant (Figure 2, 4th panel from above, right). *Tilia* seems to be more sensitive to the past weather conditions as the weights of the past meteorological elements is higher than those of the current ones except for several days before the start of pollen season when the current weather conditions become a bit more important (Figure 4, 1st panel from above, left). For *Ulmus,* again the major role of the past weather conditions can be identified except for a few days before the start of the current pollen season, when the weights of the current meteorological parameters exceed those of the past ones (Figure 4, 1st panel from above, right). The shape of the curves for *Urtica* is the most equalized of all taxa, with very similar weights of the current and past meteorological elements (Figure 4, 2nd panel from above, left).

## Discussion and conclusions

Climate change can modify the pollen season characteristics of different allergenic taxa in diverse ways and can exert a substantial influence on habitat regions. To our knowledge, a comprehensive spectrum of the regional pollen flora has yet been analysed in three studies, namely in Clot (2003, 25 plant taxa), Damialis et al. (2007, 16 plant taxa) and Cristofori et al. (2010, 63 plant taxa), respectively. The present study analyses one of the largest spectra with 19 taxa. Our study can be considered unique in the sense that it separates the effects of the current and past meteorological elements that influence the current pollen concentrations.

Airborne pollen concentrations can be influenced not only by the current values of the meteorological elements, but also by their past values. As it is hard to distinguish between the effect of the current and past values of the meteorological variables no attempt has been made so far to determine the relative weight of these two components in influencing the measured current pollen concentration. In our present approach the current meteorological elements (the daily mean temperature, daily relative humidity, daily global solar flux and daily precipitation amount) were characterised by their actual values, while past meteorological elements were described by their cumulative values. These elements were considered since they indicate the highest impact on pollen production (Galán et al. 2000; Bartková-Ščevková 2003; Štefanič et al. 2005; Kasprzyk 2008; Makra and Matyasovszky 2011). A potential importance of our procedure is that a period with a changing climate that may have a major impact on the current pollen counts can be detected by the past of meteorological elements.

The taxa examined can be classified into three groups, namely arboreal deciduous (AD), arboreal evergreen (AE) and herbaceous (H) taxa. It was found that a better comparison can be established if the taxa are separated within each group according to the starting month of their pollen season. Within the group of AD taxa, *Alnus, Populus* and *Ulmus* are marked by a definitely higher weight of the past meteorological elements in late summer – early autumn, exceeding those of the current ones almost all over the pollen-free period. For *Juglans, Morus, Platanus* and *Quercus,* the major weights of the current meteorological elements in the spring and early summer involve the most characteristic contribution to the pollen production. A few days before the start of the current pollen season the current weather conditions are predominant for all AD taxa (Figures 2, 3; Table 1). AE taxa comprise only *Pinus* and *Taxus*, but their curves are totally reverse without any similarity (Figures 2, 3; Table 1). For H taxa, Poaceae is the only taxon starting its pollination in April, so it is excluded from further analysis. Since the pollen season of *Cannabis* and Chenopodiaceae starts in early and late June, these taxa are listed into the groups pollinating from May and July, respectively. In this way, the curve of the taxa pollinating from May (*Cannabis, Plantago, Rumex* and *Urtica*) indicate the most equalized course of weights in the study. For *Cannabis, Plantago* and *Rumex* the effect of the current weather conditions is more important from the beginning of the pollen-free season, while just before the start of the new pollen season the weights of the past climate elements are more remarkable (Figures 2, 3, 4; Table 1). *Ambrosia, Artemisia* and Chenopodiaceae comprise the highest weights of the past weather conditions of all taxa until at least three months before the start of the pollination that are characteristic in determining the pollen production. However, some days or weeks before the start of the current pollen season the current climate elements have a higher importance (Figure 3; Table 1).

We should remark that stored water in the soil is much more essential to genera dominated by trees like *Quercus*, *Platanus*, *Pinus*, *Morus*, *Juglans*, *Betula* and *Alnus*. In this way, autumn and winter precipitation income better influences their growth and pollen production potentials than for herbaceous plants.

Note, that our findings for assessing the effect of the antecedent and current meteorological conditions on the current pollen concentration are valid only for variations of the daily pollen concentrations accounted for by the above-mentioned eight explanatory variables and nothing is known about the variance portion not explained by these variables.

We should remark that factor analysis with special transformation can only be applied if it is assumed that the relationships between the variables are linear. The associations analysed in the study can be non-linear, hence our results are possibly distorted. However, our methodologies are capable of separating the joint effect of the current and past weather elements respectively and, in this way; the results received can be considered as a first step towards discovering these non-linear relationships.

It should be noted that only 11-year data sets were available that involve a limitation for the analysis. The difference between the factor loadings of daily pollen counts on the current and past values of daily meteorological variables as a function of the day of the year was based on only 11-year data sets. Hence, the short data set constrains not to involve further influencing variables.

Phyto-physiological associations of the climate parameters used are as follows. Global solar flux has a major direct effect on the photosynthetic processes, as well as an indirect effect on the plants development through the temperature parameters. The start and run of the photosynthetic and generative processes occur in a limited range of temperature. In this way, extreme global solar flux involves too high or too low temperatures and, due to this, the available water may limit the vegetative and generative processes (see winter and summer periods) (Makra et al. 2011; Deák et al. 2013)*.* Concerning the four climatic parameters, the current and past components of temperature and precipitation indicate a major effect on the current pollen concentration. Long-time effect of the precipitation is manifested in the storage of groundwater or in the stored water in the plants. Relative humidity is a largely variable parameter that has a rather small long-time effect on the current pollen production. An optimum relative humidity is necessary for the plants for performing generative and vegetative (e.g. photosynthesis) processes smoothly. Relative humidity has a major role only in dry climate conditions (see Mediterranean, deserts, steppes, savannas), where the limitation or lack of groundwater is substituted partly by relative humidity. However, these conditions are not typical in the temperate belt (Láng 1998; Haraszty 2004; Makra et al. 2011; Deák et al. 2013)*.* Wind component, concerning the specific subject of the manuscript was omitted since its past values cannot be interpreted in the current pollen concentration. However, in general, wind sometimes has a substantial role in the measured local pollen counts. Depending the location of the source areas and wind direction, wind parameter can exert either positive or negative role on the local pollen level. Furthermore, long-range and medium range components of the pollen transport can also be separated and for given locations, periods of the year and pollen type their ratio is an important characteristics of the local pollen statistics (Makra et al. 2010).

We should stress that papers comparing the weight of current and past weather parameters in influencing the current local pollen production are not available in the literature. Though there are some studies presenting the impact of past weather conditions on certain phenological phases of different taxa (Spieksma et al. 1995; Giner et al. 1999; Emberlin et al. 2007, only few papers deal with this area. Additionally, no papers have been found demonstrating the influence of past meteorological elements on phenological phases of the 19 taxa analysed.

It is to be mentioned that not all possible influencing variables were considered and perhaps not the most influential variables were used in the study. However, acknowledging the limiting factors mentioned above, this is the first approach to separate the weight of the current and past weather elements in determining the current local pollen production of herbaceous and arboreal taxa.

Separation of the weight of the current and past climate conditions for different taxa presented in the study involves practical importance not only for pollen sensitized people but also for agricultural production. Namely, the knowledge of taxon specific effects of the past weather depending in time may help to predict future pollen levels well ahead in time; furthermore, it may contribute to reduce weather dependence of agricultural production.
